# Immuno-epidemiology of human *Schistosoma haematobium *infection: preferential IgG3 antibody responsiveness to a recombinant antigen dependent on age and parasite burden

**DOI:** 10.1186/1471-2334-6-96

**Published:** 2006-06-09

**Authors:** Francisca Mutapi, Takafira Mduluza, Natalia Gomez-Escobar, William F Gregory, Cecilia Fernandez, Nicholas Midzi, Rick M Maizels

**Affiliations:** 1 Institute of Immunology & Infection Research, School of Biological Sciences, University of Edinburgh, Ashworth Laboratories, King's Buildings, West Mains Rd, Edinburgh, EH9 3J, UK; 2 Department of Biochemistry, University of Zimbabwe, P.O. Box 167, Mount Pleasant, Harare, Zimbabwe; 3 National Institute of Health Research, Box CY 570, Causeway, Harare, Zimbabwe; 4 Medical Research Council, PO Box 273, Banjul, Gambia; 5 Cátedra de Inmunología, Facultad de Química, Universidad de la República, Casilla de Correo 1157, Montevideo, Uruguay

## Abstract

**Background:**

Schistosomiasis is a major parasitic disease affecting over 200 million people in the developing world with a further 400 million people at risk of infection. The aim of this study was to identify a single antigen from adult *Schistosoma haematobium *worms and subsequently use this antigen to study the development of schistosome-acquired immunity in a human population.

**Methods:**

The full-length cDNA sequence of a *S. haematobium *protein, a putative orthologue of the *S. mansoni *tegumental antigen Sm13, was obtained from a cDNA library of adult *S. haematobium *worms and named Sh13 following a small-scale expressed sequence tags (EST) project. The recombinant Sh13 protein expressed in *E. coli*, was used to investigate immuno-epidemiological patterns in 147 Zimbabweans (7–18 years old) exposed to *S. haematobium*.

**Results:**

Sequence analysis of the full-length cDNA sequence of the *S. haematobium *protein Sh13, indicated that the protein has an N-terminal signal peptide and encodes an 85-amino acid mature protein with a highly conserved predicted transmembrane domain (86 % identity with the *S. mansoni *tegumental antigen Sm13). The recombinant Sh13 protein was used in ELISA assays to determine the reactivity of sera from the study participants. Antibody responses against Sh13 were predominantly IgG3 isotype compared to responses against crude worm antigens which were predominantly IgG1 and IgG4. The relationship between anti-Sh13 IgG3 levels and infection intensity varied significantly with host age. The youngest children (7–10 years old) had relatively low levels of both infection and anti-Sh13 IgG3. In older children (11–12 years old) rising infection levels were accompanied by a significant increase in anti-Sh13 IgG3 levels. Subsequently, infection intensity declined significantly in 13–18 year olds but levels of the antibody continued to rise. The changing relationship between infection intensity and anti-Sh13 IgG3 levels with host age is consistent with the profile of a protective immune response predicted from theoretical work.

**Conclusion:**

We have identified and characterised a novel *S. haematobium *antigen Sh13, a putative tegumental protein, and shown that it is recognised predominantly by IgG3 antibodies from people infected with/exposed to *S. haematobium *parasites. We have also shown that, the anti-Sh13 IgG3 response is maximal in older individuals with the lowest infection intensity, and that the age profile of the relationship between anti-Sh13 IgG3 and infection intensity is consistent with that predicted by theoretical work for a protective response stimulated by and directed against adult worms.

## Background

Schistosomiasis is a major parasitic disease in developing countries in Africa, the Middle East, Latin America and Asia where it is estimated that 200 million people are infected with the parasite while a further 600 million are at risk of infection [1]. *Schistosoma haematobium*, the causative agent of urinary schistosomiasis occurs in 53 countries across Africa and the Middle East where it is responsible for the majority of schistosome-associated pathology. A recent survey in sub-Saharan Africa indicates that 70 million individuals out of 682 million had experienced haematuria with 32 million cases of dysuria associated with *S. haematobium *infection [2]. Furthermore, it was estimated that 18 million people suffer *S. haematobium*-related bladder wall pathology and 10 million suffer hydronephrosis [2].

Despite its public health importance, *S. haematobium *is the species least studied with respect to immune responses directed against the parasite and identification of specific antigens. In particular, few specific antigens have been identified or used for immuno-epidemiological research. In the present work, we describe the identification of a *S. haematobium *protein that, based on similarity to the already known *S. mansoni *antigen Sm13 [3], was named Sh13. Sm13 has been immunolocalised to the tegument of adult parasites and found to be recognised by total IgG from the sera of 7 Brazilians infected with *S. mansoni *[3]. The main aim of this study was to determine the immuno-epidemiological profile of anti-Sh13 responses in a Zimbabwean population exposed to *S. haematobium *infection. Use of a single antigen allows definition of response dynamics over time, which may differ markedly from those directed against other antigens.

In experimental studies, adult *S. haematobium *worms have been shown to suffer immune mediated attrition and reduction of fecundity [4-6] and, as early as 1934, Fisher postulated that the epidemiology of human schistosome infections reflects these immunological processes [7]. More recently, Woolhouse used theoretical methods to predict the profiles which these immune responses would follow in a host population [8,9] and effectively set the framework for the immuno-epidemiology of helminth infections. Since then, several field studies have been conducted in various countries to identify the immune responses responsible for these effects and to describe the immuno-epidemiology of the infection in human populations [10-18]. Identifying which of these responses are protective has been complicated by several factors, including the concurrent presence in the host of different parasite developmental stages with shared antigens, the short duration of protective responses and the limited degree of protection they provide as suggested by theoretical work [19,20]

Identifying patterns consistent with acquired immunity from field studies of infected populations has proven very difficult [21]. This is largely due to the epidemiology of infection, inherent variability in infection intensity data and the presence of factors which confound the relationship between infection levels and immune responses [8,21-23]. One useful approach is to employ results of quantitative work predicting patterns of immunity in populations, which has proved successful in other studies [14,20]. The quantitative studies predict the patterns which the immune responses should follow within the population if they are protective [8,24]. Therefore, in this study we test the predictions from quantitative work on schistosome immunity and determine if the responses to Sh13 in the study population follow these predictions.

Patterns of immunity are more likely to be detected in groups of the population showing the most pronounced changes in infection levels [8,24]. In schistosome-infected populations, children carry the heaviest parasite burdens and the greatest changes in infection levels occur in the first two decades of life [7,25]. Therefore, we focus on antibody responses of children and young adults in whom the rates of acquisition and elimination of infection are changing most rapidly. In addition, we take into account that results from the traditionally used correlation analyses across the whole population are difficult to interpret, because they arise from a heterogeneous group of people where infection dynamics differ considerably, particularly the history of infection [14]. For this reason, our study uses an age-structured analysis of the cross-sectional data which partitions the population into groups of people with similar histories of infection, enabling comparative analyses of the changing relationship between infection intensity and immune responses to be conducted. Theoretical studies and field studies in the hookworm *Necator americanus *have shown the age structured analysis to be powerful in detecting immune responses associated with protection against infection or re-infection [8,24,26].

## Methods

### Study area

The study was conducted in the Mashonaland East Province of Zimbabwe (31°30'E; 17°45'S) where S. haematobium is endemic. The study area is described in detail elsewhere [13]. Permission to conduct the work in this province was obtained from the Provincial Medical Director. The villages were selected because health surveys conducted regularly by the Provincial Medical Director in the region showed little or no infection with other helminths and a low S. mansoni prevalence (< 5%). The selected villages had not been included in the National Schistosome Control Programme and therefore had not received treatment for schistosomiasis or other helminth infections. The main activity in these villages is subsistence farming and human water contact is frequent with at least 4 contacts/person/week due to insufficient safe water and sanitation facilities (see [13] for studies in neighbouring villages). Drinking water is collected from open wells while bathing and washing is conducted in two main rivers in the villages. Most families maintain a garden located near the river where water is collected for watering the crops. The schools surveyed (a secondary school and its feeder primary school (i.e. where the majority of the primary school children come from) Goromonzi and Shangure in Goromonzi, and Chindenga and Nyambanje in Mutoko) were all in close proximity to rivers.

### Study subjects

Only permanent inhabitants of the villages who had never been treated for any helminth infection were eligible for inclusion in the study. Following explanation of the project aims and procedures to the community, school children and their teachers, an initial parasitology (stool and urine samples) and serology (blood sample) survey was conducted amongst all compliant participants. A questionnaire survey (data not shown) confirmed that on average, all participants frequented water contact sites at least four times per week and that frequency of water contacts at these sites was not significantly different within the age range included in this study. Stool samples were processed following the Kato-Katz procedure [27] to detect *S. mansoni *eggs and other intestinal helminths, while the urine filtration method [28] was used to detect *S. haematobium *eggs in urine samples. After collection of the samples, all participants were offered treatment with the recommended dose of praziquantel, 40 mg/kg body weight. In order to be included in the cohort, participants had to meet all the following criteria: 1) have provided at least 2 urine and 2 stool samples on consecutive days; 2) have given a blood sample; 3) be negative for intestinal helminths including *S. mansoni *(all people meeting criteria 1 and 2 were egg negative for intestinal helminths including *S. mansoni*, therefore, no participants were excluded on this criterion). 147 people aged between 7 and 18 years met these criteria. Serum samples obtained from 20 mls of venous blood from each participant were frozen and stored in duplicate at -20°C in the field and transferred to a -80°C freezer in the laboratory. One complete set of the samples was subsequently transported frozen from Zimbabwe to the UK, stored at -80°C and defrosted for the first time for use in this study.

### Parasites

In order to avoid any potential problem of polymorphism in antigens that may arise due to parasite strain differences, we used *S. haematobium *worms collected from a random selection of the most intensely infected participants. Eggs were hatched and used to infect snails in the laboratory in Zimbabwe. After 6 weeks, snails were shed and the cercariae thus obtained used to infect hamsters, which were sacrificed at 8 weeks post infection. To sample from a larger genetic pool, we also collected infected snails from the water contact sites used by the study population and used these to infect hamsters as above. Adult worms were recovered by perfusion of the hamsters with normal saline, washed and stored in Trizol reagent (Invitrogen) at -20°C until they were used for RNA extraction.

### Identification of Sh13

A small-scale expressed sequence tags (EST) project was conducted to generate sequences to be used in identifying antigens from a proteomics study of *S. haematobium *antigens [29]. As a result of this project a clone coding for a protein homologous to an already known *S. mansoni *adult worm antigen [3] was identified. This provided an excellent opportunity to study immune responses against the recombinant protein at a population level.

RNA was extracted from the worms stored in Trizol following the manufacturer's instructions and used to prepare a full-length enriched oligo-capped cDNA library in the pSPORT vector (Invitrogen) as published elsewhere [30]. The library was plated out and random colonies picked for EST sequencing using T7 primer and BigDye terminator sequencing ready reaction (Applied Biosystems) (see [30]). The sequences were used to search for similarities in the NCBI database. A cDNA with a translated sequence highly similar to that of the tegumental S*. mansoni *protein Sm13 [3] was thus identified and named Sh13. Because *Taq *polymerase had been used in the preparation of the cDNA library and in the generation of the ESTs, the sequence coding for the putative mature protein was confirmed by sequencing of clones derived from RT-PCR products of parasite RNA amplified with *Pfu*, a DNA polymerase with proofreading activity.

### Cloning and protein expression

Following identification of the putative full-length cDNA sequence of Sh13, the coding region was PCR amplified using specific forward and reverse primers. Because the protein has a predicted signal sequence between amino acid residues 1–19, the forward primer, which included a restriction site for *Eco*RI (underlined), was designed from residue 20: *5'-CGAGAATTCCAATCGGGACCTAGTCCAATAAAT-3'*; the reverse primer contained a site for *Sal*I (underlined) immediately after the stop codon: *5'-GCGGTCGACTTAAGATTTCTGTAAATGGTAATATAC-3'*. These primers were used to amplify the Sh13 cDNA with *Pfu *DNA polymerase from the same parasite RNA used to prepare the library. The RT-PCR product thus obtained was A-tailed with *Taq *polymerase, gel purified, ligated to pGEM^®^-T vector (Promega) and the recombinant plasmid used to transform *E. coli *JM109 cells. Inserts were then released from plasmid DNA by *Eco*RI and *Sal*I digestion, and sub-cloned into the pMal-c2x expression vector (NEB) cut with the same enzymes. Clones were verified by sequencing. The Sh13/pMal-c2x plasmid was used for protein expression following induction with IPTG according to standard protocols [31]. A control for induction and maltose binding protein (MBP) expression was also run using pMal-c2x transformants. The resulting MBP-Sh13 fusion protein was purified on an amylose column following the supplier's instructions and its identity verified by mass spectrometry. The presence of Sh13 in the fusion protein was further verified by digestion of 20 μl of MBP-Sh13 protein (1 mg/ml) with 1 íl of Factor Xa, which cleaves the arginine-isoleucine peptide bond linking MBP to Sh13. The products of this digestion were run on a gel and Coomassie blue stained.

### Antibody assays

Preliminary titrations with varying antigen concentrations, serum and secondary antibody dilutions were conducted to determine optimal conditions to carry out the enzyme-linked immunosorbent assay (ELISA) using sera from endemic normal people (i.e. heavily exposed to infection but negative for schistosome eggs), negative controls (i.e. British people who had never travelled to schistosome endemic areas) and a pool of sera from the whole population. These titration assays allowed determination of the serum dilution and antigen concentration yielding the best discrimination between negative and positive controls. The Elisa protocol was thus developed. Assays were conducted using MBP-Sh13 and MBP control as is standard [3]. ELISA plates (Nunc-Immulon, Denmark) were coated with 100 μl/well of 1 μg/ml antigen in 60 mM carbonate-bicarbonate buffer (pH 9.6) and incubated overnight at 4°C. Plates were blocked with 200 μl/well of skimmed milk (5% milk in phosphate buffered saline (PBS)/0.03% Tween 20) for 1 hr and washed three times in PBS/Tween 20, which was used for all washes. 100 μl of serum was added to each well at 1:100 dilution; plates were incubated overnight at 4°C and then washed three times. 100 μl of isotype-specific monoclonal antibody was added at 1:1000 dilution for the detection of IgA (Dako, Denmark, P0216), IgE (Vector Laboratories, UK P0720), IgG1, IgG2, IgG3, IgG4 (The Binding Site, UK, AP006, AP007, AP008 and AP009, respectively) and IgM (Dako, Denmark, P0215). Plates were incubated overnight at 4°C, washed six times and 100 μl of ABTS substrate solution (KPL, Canada) was added. The IgE-specific antibody was biotinylated; so 100 μl/well of streptavadin-horseradish peroxidase (Amersham, UK) was added at 1:6000 dilution to these plates, which were then incubated for 1 hr at 37°C, washed 6 times and developed. The reaction was allowed to take place at 37°C for 30 min for all isotypes, before the absorbance was read at 405 nm. Three negative controls used in the titration assays were included on each ELISA plate and all samples were assayed in duplicate.

For comparative purposes, antibody assays were also conducted with the conventional crude worm antigen (soluble worm antigen preparation, SWAP) prepared following standard protocols [13]. These assays were conducted following the same protocol as above using 1 μg/ml SWAP to coat the plates (a random subset of 41 samples run using 20 μg/ml SWAP together with negative and positive controls showed that similar results were obtained using 1 μg/ml and 20 μg/ml of SWAP) sera diluted at 1:100, and secondary antibodies diluted at 1:1000 for IgG1 and 1:500 for IgG2, IgG3, IgG4.

### Statistical analyses

In order to characterise the study population, preliminary analysis of variance (ANOVA) tests followed by post hoc procedures using the software package SPSS, were conducted on mean infection intensity (log_10 _(x+1) transformed) to determine the age groupings relative to infection level, i.e. age groups where infection levels are rising, peaking, and declining. After partitioning the population into the appropriate age groups, further tests were conducted on mean infection intensity (log_10 _(x+1) transformed) and antibody level (square root transformed) to determine if these changed between the age groups. Chi-squared tests were also conducted on the corresponding prevalence data for the groups. These tests partitioned the population into 4 age groups where infection levels are rising, peaking, declining and lowest.

Statistical analyses to determine factors associated with the level of antibody responses against Sh13 were conducted by ANOVA, with antibody level (square root transformed) as the dependent variable, sex (2 categories) and age (4 categories as above) as the independent variables. The interaction between age and infection intensity was also tested. The correlation between infection intensity (log_10 _(x+1) transformed) and antibody levels (square root transformed) was determined using a one-tailed Pearson correlation and the correlation coefficients thus obtained used to test for homogeneity between coefficients from the different age groups by the method of Zar [32]. The correlation analyses and homogeneity tests were also performed excluding egg negative people; the results reported here include all participants because exclusion of egg negative people did not significantly affect the results.

## Results

### Sequence analysis of Sh13

**Figure 1:**
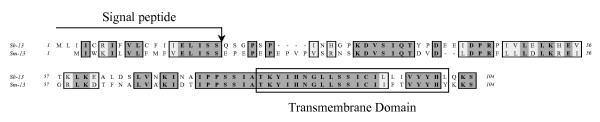
Amino acid alignment of the *S. haematobium *Sh13 and the *S. mansoni *Sm13 (accession number AAC25419) proteins.

In characterising a new cDNA library derived from adult *S. haematobium *worms we identified a clone coding for the putative orthologue of the *S. mansoni *tegumental protein Sm13 [3]. This cDNA was thus named Sh13. The full-length 312-nt ORF encodes a 104-aa protein with a theoretical mass and isoelectric point (p*I*) of 11,651 Da and 6.39 respectively calculated using ExPASy Software [33]. SignalP software [34] analysis predicted a signal peptide with a cleavage site between amino acid positions 19 and 20, which would yield a mature protein with a mass of 9,440 Da and a p*I *of 6.48. A comparison of the nucleotide sequences encoding Sm13 and Sh13 shows 70% identity; the full-length proteins are 55% identical and share a conserved *C*-terminus as illustrated in Figure 1, which has a predicted transmembrane domain [35,36] from residues 80 to 101 (in the full length sequence). The sequence is consistent with a type 1 transmembrane protein and indicates that only the final 3 residues at the *C*-terminus are cytosolic.

### Nucleotide accession number

The Sh13 cDNA sequence has been deposited in the Genbank database at the National Centre for Biotechnology Information under the bankit no 709821.

### Characterisation of recombinant Sh13

**Figure 2:**
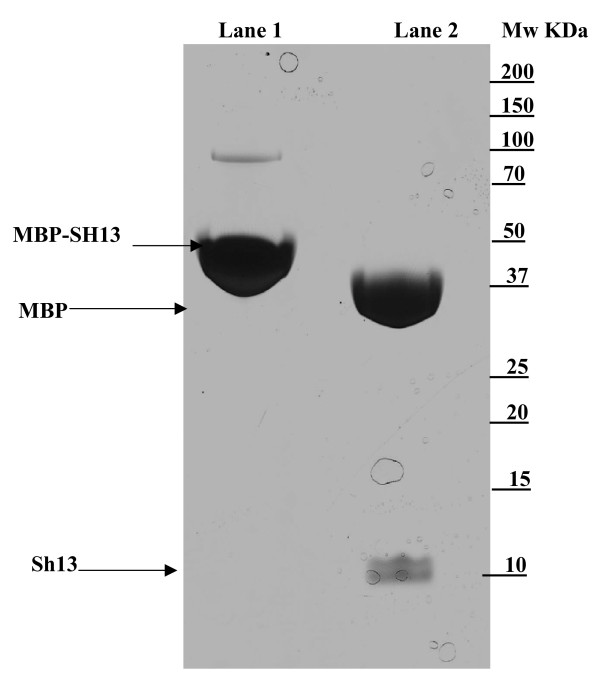
Coomassie Blue stained gel showing the Sh13-MBP fusionprotein in lane 1 and the two products; MBP and Sh13 of Factor Xa digestion in lane 2.

The sequence encoding the mature Sh13 protein was cloned into the pMal-c2x vector and the recombinant Sh13-MBP fusion expressed in *E. coli*. Mass spectrometry verified the predicted mass of the Sh13-MBP protein (52,365 Da ± 12 Da), while digestion with Factor Xa gave two products whose molecular weights were 42,490 Da ± 8, 20 Da and 9, 889 Da ±2, 25 Da (Figure 2), consistent with those predicted for MBP and the mature Sh13 respectively. Western blotting showed the protein to be recognised by the human sera used in the study as well as by sera from mice immunised with the MBP-Sh13 fusion (data not shown).

### Schistosome epidemiology in the study population

**Figure 3:**
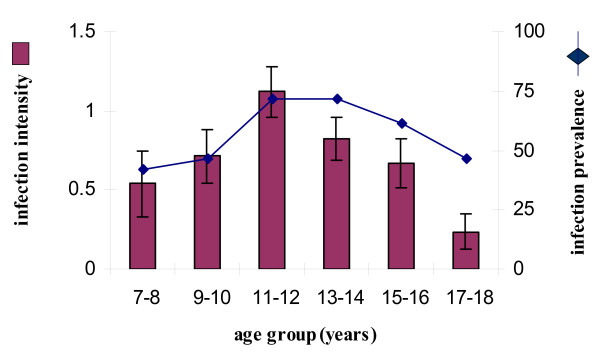
a) Infection levels of the study population divided into 2-year age classes with sample sizes as follows 7–8 years old n = 12; 9–10 years old, n = 15; 11–12 years old, n = 28; 13–14 year old, n = 46; 15–16 year old, n = 31; and 17–18 year old, n = 15. The histogram shows infection intensity (log_10_(x+1) transformed) with bars representing the standard error of the mean, while the line shows infection prevalence.

Parasitological examination gave an overall infection prevalence of 62 % and a mean infection intensity of 39 eggs/10 ml of urine (SE = 9, with a range between 0 and 1000 eggs). Infection prevalence and intensity followed a convex age-infection profile with infection levels rising to peak in children 11 to 12 years old and infection intensity declining faster than infection prevalence thereafter (Figure 3). The World Health Organisation denotes this prevalence level as high and infection intensity also as high as defined by having more than 10% of the population with more than 50 eggs/10 ml of urine (28 of the147 participants had more than 50 eggs per 10 ml urine) [37].

### Antibody responses

**Figure 4:**
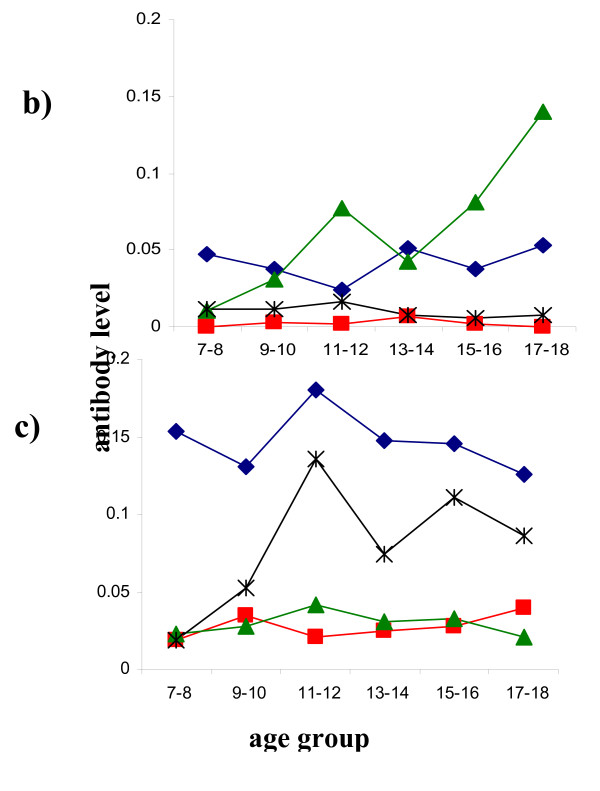
a) Age-antibody profile for anti-Sh13 IgG subclasses (square root transformed) for the age groups in figure 3 showing that IgG3 and IgG1 are the predominant subclasses produced against this antigen. Diamonds represent IgG1, squares IgG2, triangles IgG3 and asterix IgG4. b) Age-antibody profile for anti-SWAP IgG subclasses (square root transformed) for the age groups in figure 3 showing that IgG1 and IgG4 are the predominant subclasses produced against this antigen.

Initial titration assays showed that anti-Sh13 IgA, IgM and IgE could not be detected (data not shown). The lack of an anti-Sh13 IgM response also indicated that the recombinant antigen had not been contaminated by lipopolysacharides from the *E. coli *expression system. Subsequent assays focused on IgG subclasses for both anti-Sh13 and anti-SWAP responses. IgG3 was the predominant subclass produced against Sh13 while low levels of IgG1 were detectable in most people and showed little variation across the population as shown in Figure 4a. Levels of IgG2 and IgG4 were undetectable in most people and when detected, were very low. The relative levels of IgG subclasses produced against Sh13 differed from those against SWAP to which IgG1 and IgG4 responses were the predominant subclasses with little detectable IgG2 and IgG3 as shown in Figure 4b. The relationship between the predominant IgG subclasses and age also differed between the two antigen preparations, with anti-SWAP responses following the age-infection curve (Figure 4b). Further immuno-epidemiological studies focused on the anti-Sh13 IgG3, as this was the major subclass produced against the recombinant antigen, and this isotype increased markedly with age.

### Immuno-epidemiological profile of anti-Sh13 antibody responses

Statistical analysis of the relationship between the levels of anti-Sh13 and infection intensity, sex and age were conducted. Prior to this, ANOVA procedures showed that infection intensity varied significantly across the 2 year age groups shown in Figure 3 (F = 2.689, df = 5, 147, p = 0.024). Subsequent post-hoc tests showed that infection intensity in the younger age groups (7–8 years and 9–10 years, Figure 3) were similar, so these could be combined to give one age group. Similarly, infection intensity did not differ significantly between the 13–14 year olds and the 15–16 year olds where infection intensity was declining, so these groups could also be combined into one group. The oldest age group (17–18 year olds) had the lowest infection intensity which differed significant from that of 13–16 year olds, so they formed a separate age group. The study population was therefore divided into groups where infection was rising (pre- peak of infection group) – participants 7 to 10 years old (n = 27), peaking – participants 11 to 12 years old (n = 28), declining (post- peak of infection group)-participants 13–16 years old (n = 77) and lowest (lowest infection post-peak infection) – participants 17–18 years old (n = 15).

**Figure 5:**
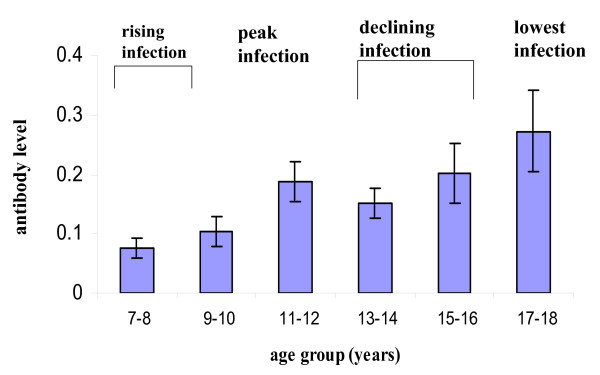
Age-antibody profile for anti-Sh13 IgG3 (square root transformed). This figure gives more detail on the anti-Sh13 IgG3 profile shown in Figure 4a, by showing the antibody levels relative to the partitioning of the population by infection intensity and also giving the standard error of the mean represented by bars on the graph.

Statistical analyses were subsequently conducted to determine if infection and antibody levels differed significantly among the four groups into which the population was partitioned and the results of these analyses are shown in Table 1. The analyses indicated that infection intensity and prevalence in the pre-peak of infection group were significantly lower that those in the peak infection group and this was matched by a significant increase in anti-Sh13 IgG3 levels Comparisons between the age groups where infection intensity peaked and declined showed a reduction albeit non significant in infection intensity and prevalence and antibody levels were largely unchanged remaining high as shown in Figure 5. However, while levels of infection continued to fall significantly as shown by the comparison between the groups with declining infection and lowest infection, anti-Sh13 IgG3 levels continued to rise. Overall these analyses demonstrated that infection levels and antibody levels rose together to peak in the 11–12 year olds, but that the subsequent decline in infection levels in 13–18 year olds was accompanied by a continued rise antibody levels as shown in Figure 5.

**Table 1:** Comparison of infection and antibody levels between the four groups.

**Age Groups**	**Infection intensity**	**Infection prevalence**	**Anti-Sh13 IgG3**
**Pre-peak vs. peak**	t = -1.942, df = 53 **p = 0.029**	χ^2 ^= 4.114, df = 1, **p = 0.043**	t = -1820, df = 53, **p = 0037**
**Pre-peak vs. declining**	t = -1.062, df = 102, p = 0.145	χ^2 ^= 4.503, df = 1, **p = 0.034**	t = -1.441, df = 102, p = 0.077
**Pre-peak vs. lowest**	t = 1.709, df = 40, **p = 0.048**	χ^2 ^= 0.019, df = 1, p = 0.890	t = -2.906, df = 40, **p = 0.003**
**Peak vs. declining**	t = 1.438, df = 103, p = 0.077	χ^2 ^= 0.145, df = 1, p = 0.704	t = 0.679, df = 103, p = 0.25
**Peak vs. lowest**	t = 3.170, df = 41, **p = 0.002**	χ^2 ^= 2.563, df = 1, p = 0.109	t = -1.165, df = 41, p = 0.126
**Declining vs. lowest**	t = 2.625, df = 90, **p = 0.005**	χ^2 ^= 2.376, df = 1, p = 0.123	t = -2.044, df = 90, **p = 0.022**

The table compares infection intensity, prevalence and anti-Sh13 IgG3 amongst the four groups which the population has been partitioned as shown in Figure 3. Results from post-hoc tests are presented after one way ANOVA showed that infection intensity and anti-sh13 IgG3 levels differed significantly across the 4 age groups (F = 3.863, df = 3, 147, p = 0.011 and F = 2.946, df = 3, 147, p = 0.045 for infection intensity and anti-sh13 IgG3 respectively). Significant differences are highlighted in bold font.

### Testing predictions from theoretical work

**Figure 6:**
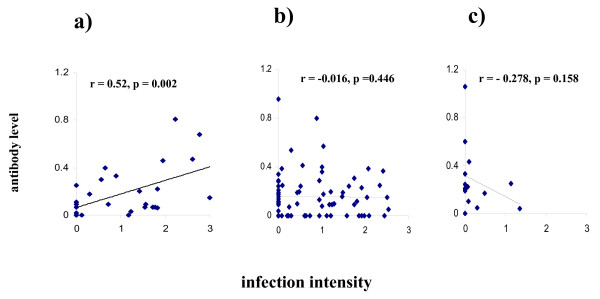
Correlation between infection intensity (log_10 _(x+1) transformed) and antibody levels (square root transformed). r is the correlation coefficient and p values are also given. The youngest age group (7–10) is excluded from the analysis as they are producing very little amounts ofanti-Sh13IgG3. a) Age group where infection levels peak (11–12 yearsold). b) Age group where infection levels are declining (13–16 years old). c) Age group where infection levels are lowest (17–18 years old).

Theoretical work modelling the age profile of a protective immune response in a helminth infection has predicted that the relationship between the immune response and infection intensity varies with host age [8,9]. Furthermore the relationship between infection intensity and the immune response will be strongest and show a positive correlation in younger age groups. This correlation is predicted to subsequently decrease turning negative in older age groups. Therefore the next step in out study was to determine if the relationship between infection intensity and anti-Sh13 levels differed between the different age groups. Detailed statistical analyses allowing for sex and age group showed that infection intensity did not have a significant effect on anti-Sh13 IgG3 levels (F = 0.329, df = 1, 147, p = 0.567). When allowing for the effects of sex and infection intensity, age group had a significant effect on anti-Sh13 levels (F = 3.971, df = 3, 147, p = 0.009). The predictions of the model were tested by analysing the effect of the interaction between age and infection intensity on anti-Sh13 IgG3 levels which was significant (F = 3.604, df = 3, 147, p = 0.015), so that the relationship between anti-Sh13 IgG3 levels and infection intensity varied depending on the age of the participant. This result meant that it was necessary to determine how this relationship changed between the different age groups. This was explored by correlation analysis in the different age groups. The youngest age group (7–10 years old) showed very little levels of anti-Sh13 IgG3 so were excluded from the correlation analyses. The analyses showed that the relationship varies between being positive and negative with the strongest, and significant correlation between infection intensity and anti-Sh13 IgG3 occurring in the age group with the highest infection intensity, the correlation subsequently reduces as shown in Figure 6. Tests for homogeneity between the correlation coefficients showed significant differences between the correlation coefficients obtained for the peak infection group and declining (post-peak of infection) (p < 0.01) and between the peak and lowest infection group (p < 0.01). Taken together these results are consistent with the predictions from the theoretical work.

## Discussion

Identification and assessment of schistosome candidate vaccine antigens has relied, and continues to rely, heavily on studies of naturally acquired immune responses in human populations [20,38,39]. However these studies have been hampered by the lack of defined single antigens, particularly for *S. haematobium*, and by methodological difficulties in detecting responses associated with resistance to infection/re-infection in field studies of human populations [40,41]. In this study, Sh13, the putative orthologue of the *S. mansoni *tegumental protein Sm13 was cloned from a cDNA library of adult schistosome worms [3]. There are no reported homologues from other organisms and as yet, there have been no functional studies of Sm13.

The recombinant Sh13 protein was recognised by mouse sera following immunisation (data not shown) and was reactive predominantly with IgG3 and also with IgG1 antibodies in sera from the Zimbabwean population exposed to *S. haematobium *infection. The pattern of reactivity of the sera against Sh13 differed from that against adult worm crude antigens, where the predominant isotypes were IgG1 and IgG4. Responses to crude antigens are more heterogeneous as they are directed against multiple epitopes present on numerous uncharacterised antigens and therefore may mask the relationship between infection levels and responses to specific antigens [40].

The epidemiology of schistosome infection in this population followed a convex shape of rising infection levels (intensity and prevalence) peaking in childhood and declining with age thereafter, a pattern which has been attributed to cumulative exposure to infection and the development of gradually acquired resistance to schistosomes [8,11,14,20,42]. The relationship between infection intensity and anti-Sh13IgG3 levels varied significantly with age. The low levels of infection intensity in the youngest age group were matched by low levels of the antibody, presumably reflecting a low cumulative exposure to adult parasite antigens in these young children. This is consistent with an earlier report on children resident in a *S. haematobium *endemic area in Zimbabwe showing that the IgG3 response against adult worm antigens developed slowly in young children 4 months- 6 years old [43]. Infection intensity and prevalence increased significantly to peak in children 11–12 years old. This significant change in infection levels was matched by a significant increase in anti-Sh13IgG3. Correlation analysis showed a significant positive association between infection intensity and anti-Sh13IgG3 levels in the age group presenting the highest egg counts allowing for the conjecture that the anti-Sh13 IgG3 response is stimulated by the increasing parasite burden. While infection intensity declined significantly in the oldest age group, anti-Sh13 IgG3 levels remained high so that the relationship between levels of this response and infection intensity is uncoupled as illustrated by the changes in the correlation between infection intensity and levels of the antibody in this age group.

The changing relationship between anti-Sh13 IgG3 and infection intensity with host age observed here was confirmed by the significant change in the correlation coefficient between the two variables with host age, and is consistent with that predicted for immune responses associated with resistance to infection [8]. Theoretical work following the profile of a protective immune response triggered by and directed against antigens in the adult worms in a population, younger age groups as observed here [8]. Furthermore, this correlation is predicted to fall in age groups with declining infection levels, so that a significant change in correlation coefficients with age is expected to occur as observed here. Similar changes have previously been reported in immuno-epidemiological studies of *Necator americanus *IgG responses against adult worm excretory/secretory antigens [44], and interpreted as suggesting the development of protective parasite-specific acquired immunity [8,44].

There are several competing explanations for the falling infection intensity/prevalence and uncoupling of *S. haematobium *infection intensity and antibody levels observed here. These include changes in exposure to infection resulting in lower worm burdens and physiological changes resulting in lower susceptibility to infection. In this population data from the initial questionnaire studies did not show a significant change in exposure to infective water throughout the age range of the participants. In addition, our previous studies and those of other groups working on *S. haematobium *show that reduction in water contact with increasing age is in itself insufficient explanation for the observed decline in infection levels [14,45-48]. Age-related physiological changes have also been suggested as a possible explanation for the development of innate resistance to infection, but evidence for this from field studies is still lacking. The alternative explanation is the development of schistosome-specific acquired immunity, [14,20,42] and the patterns of infection and antibody responses in our study are consistent with the effects of acquired immunity against the parasite.

If the expression pattern of Sh13 is similar to that of Sm13, which is expressed only in adult worms [3], then the anti-Sh13 response is being elicited by adult worms (both living and dying) and is directed against adult worms. In *S. mansoni*, the protein has been immunolocalised to the tegument and therefore is accessible to the host's immune system. Work to characterise the expression of Sh13 is now underway. From the present work, we can conclude that the profile of anti-Sh13 IgG3 is consistent with that of a protective response, but we have yet to establish that the anti-Sh13 response contributes to, rather than simply, reflects the development protective immunity against schistosome infections. We have obtained encouraging results from our preliminary vaccination studies in 10 hamsters which have shown that Sh13 stimulates an antibody response in hamsters and that vaccinated hamsters have little or no infection compared to unvaccinated controls which had heavy infection and liver pathology (data not shown). These studies are now being repeated using a larger sample size. Clearly, protective immunity will be multifaceted involving different cellular and humoral responses to several antigens.

## Conclusion

We have identified and characterised a novel *S. haematobium *antigen Sh13, a putative tegumental protein, and shown that it is recognised predominantly by IgG3 antibodies from people infected with/exposed to *S. haematobium *parasites. We have also shown that, the anti-Sh13 IgG3 response is maximal in older individuals with the lowest infection intensity, and that the age profile of the relationship between anti-Sh13 IgG3 and infection intensity is consistent with that predicted by theoretical work for a protective response stimulated by and directed against adult worms.

## Competing interests

The author(s) declare that they have no competing interests.

## Authors' contributions

FM conducted the field work, prepared the cDNA library, expressed the recombinant protein, conducted immunology assays, analyzed the data and drafted the manuscript. All these stages received significant contributions from all the other authors as follows: TM and NM conducted field work collecting parasitology and immunology samples from the participants as well as collecting parasite material and maintaining the life cycle to prepare the cDNA. CF lead the cDNA library preparation, NG-E lead the protein expression; WBG analyzed the sequences, conducted the alignment and characterized Sh13. The overall study was overseen by RMM who also designed primers for Sh13 protein expression and helped with the preparation of the manuscript. All authors read and corrected draft copies of the manuscript and approved the final version.

## Pre-publication history

The pre-publication history for this paper can be accessed here:


